# Quantifying the human vaginal community state types (CSTs) with the species specificity index

**DOI:** 10.7717/peerj.3366

**Published:** 2017-06-27

**Authors:** Zhanshan (Sam) Ma, Lianwei Li

**Affiliations:** Computational Biology and Medical Ecology Lab, State Key Laboratory of Genetic Resources and Evolution, Kunming Institute of Zoology, Chinese Academy of Sciences, Kunming, Yunnan, China

**Keywords:** Species specificity, Community diversity, Community state type (CST), Human vaginal microbial community, Specificity aggregation index (SAI)

## Abstract

The five community state types (CSTs) first identified by Ravel et al. (2011) offered a powerful scheme to classify the states of human vaginal microbial communities (HVMC). The classification is a significant advance because it devised an effective handle to deal with the enormous inter-subject heterogeneity and/or intra-subject temporal variability, the quantification of which is extremely difficult but of critical importance such as the understanding of BV (bacterial vaginosis) etiology. Indeed, arguably the most plausible ecological hypothesis for interpreting the BV etiology heavily depends on the CST classification (Gajer et al., 2012; Ma, Forney & Ravel, 2012; Ravel et al., 2011). Nevertheless, the current form of CSTs is still qualitative and lacks a quantitative criterion to determine the CSTs. In this article, we develop a quantitative tool that can reliably distinguish the CSTs by applying the *species specificity* of Mariadassou, Pichon & Ebert (2015) and the *specificity aggregation index* (SAI) we propose in this study. The new tool accurately characterized the classifications of the five CSTs with both 400-crosssectional cohort (Ravel et al., 2011) and 32-longitudinal cohort (Gajer et al., 2012) studies originally utilized to develop the CST scheme. Furthermore, it offers a mechanistic interpretation of the original CST scheme by invoking the paradigm of specificity continuum for species adaptation and distribution. The advances we made may not only facilitate the accurate applications of the CST scheme, but also offer hints towards an effective tool for microbiome typing such as classifying gut enterotypes.

## Introduction

Based on a cross-sectional study of 394 healthy women at reproductive ages, Ravel et al. (2011) classified the human vaginal microbial communities (HVMC) into five community state types (CSTs). Specifically, CSTs I, II, III, and V are dominated by *L. crispatus*, *L. gasseri*, *L. iners*, and *L. jensenii* respectively, i.e., all four groups are dominated by *Lactobacillus* spp. CST IV has no specific dominant species and was termed *diverse* group. Type IV was characterized by higher proportions of strictly anaerobic bacteria including *Prevotella, Dialister, Atopobium, Gardnerella, Megasphaera, Peptoniphilus, Sneathia, Eggerthella, Aerococcus, Finegoldia,* and *Mobiluncus*. A signature of CST-IV is higher community evenness due to the lack of dominant species.

In a later longitudinal study of 32 healthy women at reproductive ages, Gajer et al. (2012) further classified HVMC CST IV into type CST IV-A and IV-B. The former is characterized by modest proportions of either *L. inners* or other *Lactobacillus spp*, together with low proportions of various species of strictly anaerobic bacteria such as *Anaerococcus, Corynebacterium, Finegoldia*, or *Streptococcus*. In contrast, the latter state type IV-B is characterized by higher proportions of the genus *Atopobium*, in addition to *Prevotella, Parvimonas, Sneathia, Gardnerella, Mobiluncus*, or *Peptoniphilus* and several other taxa. Another distinction between CST IV-A and CST IV-B is that the latter contains some of the BV associated bacteria (BVAB) and is often associated with high Nugent scores, while CST IV-A is often associated with low Nugent scores. It is noted that, perhaps due to relatively small sample size (compared with the previous cross-sectional study of nearly 400 individuals) CST-V was not detected in the longitudinal study. Table 1 in the section of ‘Materials and Methods’ shows the classification of the five types based on the cross-sectional (Ravel et al., 2011) and longitudinal (Gajer et al., 2012) studies, including two subtypes (IV-A & IV-B) of the diversity group (CST IV).

**Table 1:** The datasets utilized to define the HVMC community state types (Ravel et al., 2011; Gajer et al., 2012) and also to test our quantification with the species specificity index.

Dataset	400 cross-sectional cohort				
Types	Type I	Type II	Type III	Type IV	Type V
Dominant species	*L. crispatus*	*L. gasseri*	*L. iners*	N/A	*L. jensenii*
Number of samples	105	25	135	108	21

Dataset	32 longitudinal cohort					
Types	Type I	Type II	Type III	Type IV		Type V
				IV-A	IV-B	
Dominant species	*L. crispatus*	*L. gasseri*	*L. iners*	N/A	N/A	*L. jensenii*
Number of samples	163	57	379	91	247	0

The characterization of the HVMC into five types by Ravel et al. (2011) is a very significant advance towards better understanding of the HVMC, yet the issue is far more complex than on the surface. The finding is significant, at least, for the following three reasons. Firstly, HVMCs are heterogeneous at various spatial (inter-individual, inter-ethnic groups) and temporal (intra-individual across time) scales; yet, characterizing them turned out to be rather elusive. The five-type classification scheme seems to be the most robust and consistent characterization available to date (Ravel et al., 2011; Gajer et al., 2012; Ma, Forney & Ravel, 2012; MacIntyre et al., 2015). For example, although the inter-ethnic group difference in the community composition does exist (e.g., Ravel et al., 2011; Gajer et al., 2012; Ma, Forney & Ravel, 2012), the commonalities between ethnic groups seem to be far more significant than their differences, and the distinction between CSTs is far more conspicuous and consistent than the difference between various ethnic groups (Ravel et al., 2011). Secondly, although the intra-subject or temporal heterogeneity of the HVMC is rather dramatic, Gajer et al. (2012) demonstrated that the most effective characterization of the temporal dynamics was still the CSTs i.e., the HVMC of a woman is a dynamic system with the transitions of the CTS. In other words, *typing* was still the fundamental element for describing the temporal dynamics of the HVMCs. For this reason, Gajer et al. (2012) emphasized the necessity to make distinctions among *community state types* (CSTs) (i.e., types of community state), community states (which are dynamic and may change over time), and community class (i.e., set of community states). Thirdly, the concept of CST also provides an important tool to investigate the etiology of BV (bacterial vaginosis) and to conduct personalized diagnosis and treatment of BV.

The complexity of community state typing is two-fold. On the one hand, the complexity is inherent with the problem of characterizing the HVMC *per se*, i.e., the enormous spatial and/or temporal heterogeneities mentioned previously. On the other hand, the original community-state typing was based on sophisticated data analysis; specifically, CSTs are clusters of community states derived from hierarchical clustering using Ward linkage and Jensen–Shannon divergence dissimilarity measure. The extremely fluctuation nature of microbial population abundance further complicates the community typing problem, by imposing the complexity on top of the enormous heterogeneities both spatially and temporarily, as stated previously. In this article, we report a quantitative approach for characterizing the CST by applying the *species specificity* measure, proposed recently by Mariadassou, Pichon & Ebert (2015). The concept of *specificity* is based on the well-known specialist-generalist paradigm, which predicts that a specialist should have a local advantage over a generalist and hence be more abundant (Mariadassou, Pichon & Ebert, 2015). Mariadassou, Pichon & Ebert (2015) devised the *species specificity* index based on a reinterpretation of the *indicator* values of Dufrene & Legendre (1997) and applied the newly reincarnated measure to analyze *abundance-specificity* relationships in microbial ecosystems. A major finding from their application of the specificity index to the microbial ecosystems is that microbial habitats are consistently dominated by *specialist* taxa, leading to a strong and *positive* correlation between *abundance* and *specificity*. In the present study, we leverage the power of the specificity index in characterizing the *habitat-specific* specialist taxa for quantifying the CSTs of the human vaginal microbial communities.

## Material and Methods

### The five community state types (CSTs) of the human vaginal microbiome and relevant datasets

As briefly reviewed in the ‘Introduction’, the five CSTs, first proposed by Ravel et al. (2011) and later supplemented by Gajer et al. (2012), include five major types and two sub-types under CST IV, i.e., CST IV-A and CST IV-B. These five types can be distinguished as two categories, one category including CST I, II, III, and V. This category of CSTs is dominated by *Lactobacillus* spp., but each type has its uniquely different dominant species (see Table 1). We term the first category *Lactobacillus* dominated types (LDT). The second category includes CST IV only and lacks conspicuous dominant species. CST IV was termed *diverse* group (type) and was further classified as CST IV-A and CST IV-B (Gajer et al., 2012). The distinction between CST IV-A and IV-B can be summarized as two points: (i) CST IV-A consists of moderate proportions of either *L. inners* or other *Lactobacillus spp*, plus low proportions of various species of strictly anaerobic bacteria such as *Anaerococcus, Corynebacterium, Finegoldia*, or *Streptococcus*. In contrast, CST IV-B is characterized by higher proportions of the genus *Atopobium*, together with *Prevotella, Parvimonas, Sneathia, Gardnerella, Mobiluncus*, or *Peptoniphilus* and several other taxa. (ii) CST IV-A often has low Nugent score, and type IV-B includes some of the BV associated bacteria (BVAV) and is often associated with high Nugent scores. The following table shows the classification of CSTs in the cross-sectional study of 394 subjects (Ravel et al., 2011), which we term as 400-cross-sectional cohort, as well as in the longitudinal study of 32 subjects (Gajer et al., 2012) studies, which we term as 32-longitudinal cohort in this report. A very brief description for both datasets is provided below.

Ravel et al.’s (2011) cross-sectional cohort study obtained 16s-rRNA sequence data of the vaginal microbial communities from 396 women representing four ethnic groups (98 white, 104 black, 97 Asian, and 97 Hispanic). Each processed 16S rRNA gene sequence was classified at a genus level using the Ribosomal Database Project (RDP) Naive Bayesian Classifier and further refined with SpeciateIT (speciateIT.sourceforge.net) to the species level. The resulting OTU table is available online as Table S4 (www.pnas.org/lookup/suppl/doi:10.1073/pnas.1002611107/-/DCSupplemental/st04.xlsx) of the original publication (Ravel et al., 2011). Gajer et al. (2012) longitudinal cohort study obtained 16s-rRNA sequence data of the vaginal microbial communities of 32 women during a three-month period (each woman was sampled approximately 30 times during the period). The data analysis and OTU assignments were similar to the procedures briefly mentioned above for the cross-sectional study and described in details in Gajer et al. (2012). The resulting OTU table is available as the online supplementary material (http://stm.sciencemag.org/content/4/132/132ra52.full) of Gajer et al. (2012) original publication. The present study is based on a reanalysis of these two sets of OTU tables mentioned previously.

### Specificity continuum

Mariadassou, Pichon & Ebert (2015) defined the *specificity*, in a spatial setting (such as habitat locations) as a measure for the *unevenness* (*heterogeneity*) with which a taxon occurs in different *habitats*. A significance of the specificity concept is to reflect the differences among habitats in the *abundance-rank distributions* (i.e., *species abundance distributions*). The *specificity continuum* can be considered as a reincarnation of the *specialist-generalist* paradigm, which has been extensively investigated traditionally in ecology. The specificity continuum of Mariadassou, Pichon & Ebert (2015) identified two extremes corresponding to the entities in the traditional specialist-generalist paradigm: (i) taxa detected with equal abundances in many habitats, i.e.,* generalists* and (ii) taxa always and only detected in one habitat, i.e., *specialists*. A subtle difference between the *specificity continuum* and traditional *generalist-specialist* paradigm is that the former tries to describe the relationship between specificity and relative local abundance in different microbiota habitats, defined by *environment type*, rather than *locality*, stressing that habitats may be affected by diverse biotic and abiotic environmental factors (Mariadassou, Pichon & Ebert, 2015). In the context of CST characterization in this study, we postulate that there are CSTs (similar to the environment types or host types mentioned above) that may be influenced by diverse biotic and abiotic host factors.

One central goal Mariadassou, Pichon & Ebert (2015) accomplished was to test whether or not the positive correlation relationship of *abundance-specificity* is prevalent in microbial ecosystems, and their tests with extensive datasets, chosen to represent a wide array of environments, habitats, sampling conditions, and sequencing depths, confirmed the strong, positive relationships between species specificity and their local abundances. Besides leveraging this important finding by Mariadassou, Pichon & Ebert (2015), we are particularly interested in exploring utilizing specificity measure to quantify the five CSTs in the human vaginal microbiome. In fact, extreme specialists can act as *indicator species* in the community, with strong ecological preferences, which are specific to a given habitat (Dufrene & Legendre, 1997; Mariadassou, Pichon & Ebert, 2015), or CST in our case.

In the following, we briefly outline the definition and computation procedure of the specificity index recently developed by Mariadassou, Pichon & Ebert (2015), which will be applied to quantify the CSTs of the human vaginal microbiome with the 400-cross-sectional cohort dataset (Ravel et al., 2011) and 32-longitudinal cohort dataset (Gajer et al., 2012).

Mariadassou, Pichon & Ebert (2015) specificity index is a reinterpretation of Dufrene & Legendre’s (1997) indicator values. Let *M* = (*a*_*ij*_) be the OTU table representing the composition of a microbiota, where *a*_*ij*_ is the relative abundance of species *i* in sample *j*; *H* be the number of different habitats (e.g., different hosts); *S*^*h*^ be the number of samples from habitat *h*; ${S}_{i}^{h}$ be the number of samples from habitat *h* where species *i* is present. The local specificity index

(1) \begin{eqnarray*}{\mathop{\wedge \nolimits }\nolimits }_{i}^{h}={A}_{i}^{h}\times {B}_{i}^{h}\end{eqnarray*}

where ${A}_{i}^{h}= \frac{{S}_{i}^{h}}{{S}^{h}} $, ${B}_{i}^{h}= \frac{{ \left\langle {a}_{i} \right\rangle }^{h}}{{\mathop{\sum }\nolimits }_{h=1}^{H}{ \left\langle {a}_{i} \right\rangle }^{h}} $ and ${ \left\langle {\text{a}}_{\text{i}} \right\rangle }^{h}= \frac{{\mathop{\sum }\nolimits }_{j=1}^{{S}_{h}}{a}_{ij}}{{S}^{h}} $.

Obviously, ${A}_{i}^{h}$ is the *prevalence* of species *i* in habitat *h*, i.e., the fraction of samples from habitat *h* where species *i* was found. ${ \left\langle {a}_{i} \right\rangle }^{h}$ denotes the average local abundances of species *i* in habitat *h*, and ${B}_{i}^{h}$ denotes the share of habitat *h* in the total population of species *i*.

Note ${\mathop{\wedge }\nolimits }_{i}^{h}\in [0,1]$, a value of zero indicates that the species is absent in habitat *h*, while a value of *1* indicates that species is always detected and only detected in that habitat and therefore a perfect indicator of that habitat.

We postulate that the dominant species in the LDT or CSTs I, II, III and V should have the highest *prevalence* across individuals in their respective CST, i.e., a *habitat* or *environment* (host) type in terms of Mariadassou, Pichon & Ebert (2015) specificity concept. They should also have the highest *share* in their respective local communities in terms of the population abundance. Accordingly, those dominant species should have the highest specificity value in their respective CSTs. As to the CST IV, we postulate that none of the species in this type should have predominantly large specificity value, and the distribution of their specificity values should be rather *dispersed* (even or less aggregated). We further devise a simple *specificity aggregation index* (SAI) (Eq. (2)) to measure the *aggregation* or *dispersion* of specificity, and to facilitate the quantification of the CSTs. In the following, we test this postulation with both 400 cross-sectional cohort and 32 longitudinal cohort datasets, and further discuss its potential biomedical implications.

The *specificity aggregation index* (SAI) is defined as:

(2) \begin{eqnarray*}SAI={V}_{S}/{M}_{S}\end{eqnarray*}

where *M*_s_ is the *mean* of specificity values of the top 10 species (OTUs) with highest specificity values, and *V*_s_ is the corresponding variance. We expect that the SAI of CST-IV should have rather low aggregation (high dispersion or evenness), compared with the other four types.

### Community diversity profiles in the Hill numbers

To further explore the medical ecology implications of the CSTs, we computed their community *diversity profiles* with the Hill numbers by applying the recent advances in measuring biodiversity. The Hill numbers, originally introduced as an *evenness* index from economics by Hill (1973) who was apparently inspired by Reny’s (1961) general entropy of order, has not received the attention it deserves in ecology until recent years. Jost (2007) and Chao, Chiu & Hsieh (2012) further clarified Hill’s numbers for measuring alpha diversity as:

(3) \begin{eqnarray*}{\text{}}^{q}D={ \left( \sum _{i=1}^{S}{p}_{i}^{q} \right) }^{1/(1-q)}\end{eqnarray*}

where *S* is the number of species, *p*_i_ is the relative abundance of species *i*, *q* is the order number of diversity.

The Hill number is undefined for *q* = 1, but its limit as *q* approaches to *1* exists in the following form:

(4) \begin{eqnarray*}{\text{}}^{1}D=\lim _{q\rightarrow 1}{\text{}}^{q}D=\exp \nolimits \left( -\sum _{i=1}^{s}{p}_{i}\log \nolimits ({p}_{1}) \right) .\end{eqnarray*}

The ^*q*^*D* offers a series of entropy values (Hill numbers) at different nonlinearity levels (i.e., diversity order *q*) and is termed *diversity profile*. The *diversity profile* is similar to the *moments* in statistics. In statistics, the zero-*th* moment is the total probability (i.e., one), the first-order moment is the arithmetic *mean*. The variance, kurtosis, and skewness are centered 2nd, 3rd and 4th order moments, respectively. For most probability distributions, the collection of all the moments of all orders (*q* = 0, 1, 2, 3, …) uniquely determines the probability distribution.

Similarly, the collection of all the Hill numbers of all orders uniquely determines the species abundance distribution (SAD) of a community, which fully describes the community composition. The parameter *q* determines the *sensitivity* of the Hill number to the relative frequencies of species abundances. The Hill numbers of the zero-*th* order (*q* = 0) is the *species richness* (i.e., ^0^*D* = *S*); in this case, the species abundances (frequencies) do not contribute to the sum in Eq. (1). The first-order (*q* =1) Hill numbers is the exponential of the Shannon entropy (Eq. (4)); in the first order, the Hill numbers weigh species in proportion to their frequency and represent the number of ‘*typical*’ species. The second-order (*q* = 2) Hill numbers is the reciprocal of Simpson index, i.e.,

(5) \begin{eqnarray*}{\text{}}^{2}D= \left( 1/\sum _{i=1}^{S}{p}_{i}^{2} \right) .\end{eqnarray*}

In the second order, Hill numbers weigh species in favor of abundant species and discount rare species; ^2^*D* therefore represents the number of dominant species.

The general interpretation of diversity of order *q* is that the community contains ^*q*^*D* = *x* equally abundant species, which is why Hill numbers are referred to as *effective numbers of species* or as *species equivalents*.

It is interesting to note the contrasting difference between *species specificity* and *community diversity*. As mentioned previously, the former captures the unevenness (heterogeneity) of a species across different habitats, and the later obviously measures the unevenness of all species in a community (a habitat). While community diversity is well established in community ecology, and it indeed may be utilized to distinguish between LDT and CST IV, it cannot delineate all five CSTs. In contrast, Mariadassou, Pichon & Ebert (2015) specificity metric can, with the assistance of our SAI, accomplish the task readily.

### Statistical significance tests and distribution-fittings

We use non-parametric Mann–Whitney rank-sum statistic to test various hypotheses regarding the difference in community diversity and species specificity among various CSTs. We further investigate the statistical distributions of the diversity and specificity, respectively, by fitting two contrastingly different statistical distributions: the normal distribution and power-law distribution. For example, the power-law distribution usually reveals heavy heterogeneity, and the heterogeneity can be so high that the *average* of the distribution cannot represent most data points in the distribution (the so-called “*no average*” property). Such information should be particular valuable for further characterizing the CSTs and understanding their medical ecology implications.

**Table 2:** The top 10 species (OTU) with the highest specificity value in the five CSTs of the 400-crosssectional cohort.

Type I		Type II		Type III		Type IV		Type V	
Species	Specificity	Species	Specificity	Species	Specificity	Species	Specificity	Species	Specificity
*L. crispatus*	0.952	*L. gasseri*	0.923	*L. iners*	0.778	*Prevotella*	0.783	*L. jensenii*	0.915
*Lactobacillales_6*	0.944	*Lactobacillales_1*	0.710	*Lactobacillales_2*	0.724	*Dialister*	0.750	*Lactobacillales_5*	0.456
*Clostridium*	0.114	*L.vaginalis*	0.369	*Lactobacillales_5*	0.293	*Atopobium*	0.678	*Lactobacillales_7*	0.243
*Lactobacillus_2*	0.099	*Anaerococcus*	0.244	*Finegoldia*	0.036	*Eggerthella*	0.664	*Propionibacterium*	0.203
*Staphylococcus*	0.083	*Peptoniphilus*	0.214	*Staphylococcus*	0.035	*Sneathia*	0.662	*Streptococcus*	0.122
*L.vaginalis*	0.077	*Lactobacillus_3*	0.155	*Ureaplasma*	0.034	*Parvimonas*	0.659	*Enhydrobacter*	0.087
*Lactobacillales_5*	0.070	*Gardnerella*	0.153	*Corynebacterium*	0.032	*Ruminococcaceae_3*	0.655	*Corynebacterium*	0.070
*L.iners*	0.060	*Finegoldia*	0.149	*Aerococcus*	0.031	*Megasphaera*	0.655	*Acinetobacter*	0.056
*Lactobacillales_2*	0.033	*Bifidobacterium*	0.144	*Lactobacillales_7*	0.026	*Prevotellaceae_2*	0.601	*Finegoldia*	0.050
*Exiguobacterium*	0.031	*Ureaplasma*	0.114	*Lactobacillus_2*	0.025	*Mobiluncus*	0.504	*Skermanella*	0.048
**Mean (M)**	0.246		0.318		0.201		0.661		0.225
**Variance (V)**	0.137		0.077		0.091		0.006		0.075
**SAI**=**V/M**	0.558		0.242		0.451		0.009		0.333
**SAI ratio to type IV**	62		27		50		1		37

Since the information on normal distribution can be readily found in standard statistics textbook (e.g., Gotelli & Ellison, 2013), we only list some basic information about the power law distribution below. Power law distribution has a probability density function as follows:

(6) \begin{eqnarray*}p(x)= \frac{K-1}{{x}_{\mathrm{min}}} { \left( \frac{x}{{x}_{\mathrm{min}}} \right) }^{-K}\end{eqnarray*}

where *x* is the random variable, *x*_min_ is the minimum value of *x*, and *K* is the exponent of the power law distribution, which has rich information about heterogeneity of the distribution. A comprehensive discussion on the power law distribution, including its fitting to data, can be found in Clauser, Shalizi & Newman (2009).

## Results and Discussion

### Testing the CST quantification with the 400-cross-sectional cohort dataset

The specificity values of the top 10 species (OTUs) with highest specificity for each CST in the 400 cross-sectional cohort are listed in Table 2, and the corresponding results of all species for all CSTs in the cohort are listed in Table S1. As expected, in the LDT or CST I, II, III, and V, the *dominant* species in each type indeed exhibited the highest specificity values in their respective CST. Specifically, the dominant species *L. crispatus, L. gasseri, L. iners,* and *L. jensenii* command the top position with a series of specificity value of 0.952, 0.923, 0.778, and 0.915 in their respective CST I, II, III and V. In CST IV, the specificity values of top 10 species are rather dispersed (less aggregated) with a range from 0.504 to 0.783, which is far less aggregated than the range in the other four CSTs (the most aggregated in the other four groups ranges from 0.114 to 0.923), besides lacking a predominant ‘leader’ as in CST I, II, III, and V.

The specificity aggregation index (SAI) values for the five CSTs are 0.558, 0.242, 0.451, 0.009, and 0.333, respectively, as displayed in Table 1. The SAI for type IV is significantly smaller than the SAI values of the other four CSTs. The contrasting difference between CST IV and other CSTs is even more conspicuous from another index, the ratio to CST IV, which is the SAI of the other CSTs divided by the SAI of CST-IV, and the ratios are 62, 27, 50, and 37 for CST I, II, III, and V respectively. Of course, the ratio to CST IV of itself is the unit *1*. This indicates that the other four CSTs are, to the minimum, 27 times more aggregated than CST IV. In other words, the SAI of CST IV is far more dispersed (even) and lacks a dominant species, which is of course consistent with Ravel et al.’s (2011) original definition of the CSTs.

As demonstrated in the following ‘Discussion’, although diversity analysis, especially with diversity profile in the Hill numbers can successfully distinguish between CST IV and other four CSTs, i.e., the LDT, it cannot further distinguish the other four types (i.e., I, II, III, and V). In contrast, the specificity index in combination with our simple SAI can successfully distinguish all five CSTs. In the next sub-section, we test our quantification approach with the 32-longitudinal cohort dataset, in which Gajer et al. (2012) further classified Type V into type IV-A and IV-B.

### Testing the CST quantification with the 32-longitudinal cohort dataset

To further test our SAI-based CST quantification scheme with the 32-longitudinal cohort dataset, we pooled together all 937 samples from 32 individuals and reclassify them into five types (including two subtypes of CST IV) according to Gajer et al. (2012) classification of those community samples, i.e., CST I, II, III, IV-A, and IV-B. It should be noted that the concept of CST is neither specific to ethnic group, nor to individual. Instead, it is specific to community sample, or a snapshot of a community, the very reason it was termed *community state type* (Ravel et al., 2011; Gajer et al., 2012; Ma, Forney & Ravel, 2012). Hence, the pooling of community sample across individuals is not only justified but also necessary to test the quantification here. Table 3 lists the 10 species with top 10 highest specificity values for each CST, and the full results of all species in the 32-longitudinal cohort dataset are listed in Table S2.

**Table 3:** The top ten species (OTU) with the highest specificity value in the five types of 32 longitudinal cohort.

Type I		Type II		Type III		Type IV-A		Type IV-B	
Species	Specificity	Species	Specificity	Species	Specificity	Species	Specificity	Species	Specificity
*L.crispatus*	0.848	*L.gasseri*	0.981	*L.iners*	0.788	*Anaerococcus*	0.752	*Atopobium*	0.908
*L.otu3*	0.173	*L.otu4*	0.968	*L.otu5*	0.777	*Finegoldia*	0.712	*Coriobacteriaceae.3*	0.695
*L.jensenii*	0.157	*L.otu1*	0.688	*L.jensenii*	0.354	*Corynebacterium*	0.639	*Gardnerella*	0.660
*L.reuteri*	0.156	*L.otu2*	0.497	*Lactobacillales.2*	0.293	*Peptoniphilus*	0.612	*Sneathia*	0.655
*L.vaginalis*	0.102	*Ureaplasma*	0.324	*L.otu3*	0.169	*Streptococcus*	0.570	*Parvimonas*	0.606
*Alloscardovia*	0.066	*Arthrobacter*	0.301	*Aerococcus*	0.070	*Incertae_Sedis_XI.1*	0.563	*Ruminococcaceae.3*	0.587
*Bifidobacterium*	0.054	*Brevibacterium*	0.297	*Ureaplasma*	0.068	*Porphyromonas*	0.530	*Mobiluncus*	0.447
*Archaea.7*	0.053	*Weissella*	0.179	*Gardnerella*	0.044	*Campylobacter*	0.518	*Aerococcus*	0.431
*Dialister*	0.033	*Corynebacterium*	0.124	*L. vaginalis*	0.040	*Incertae_Sedis_XI.2*	0.503	*Allisonella*	0.421
*Propionimicrobium*	0.029	*L.vaginalis*	0.115	*L. plantarum.*	0.029	*Prevotella*	0.465	*Megasphaera*	0.411
**Mean (M)**	0.167		0.447		0.263		0.586		0.582
**Variance (V)**	0.060		0.107		0.087		0.009		0.025
**SAI**=**V/M**	0.360		0.239		0.332		0.015		0.043
**SAI ratio to IV-A**	24		16		22		1		2.9
**SAI Ratio to IV-B**	8		5		8		0.3		1

Similar to the finding demonstrated in the previous 400 cross-sectional cohort dataset, the results with the 32-longitudinal cohort dataset displayed in Table 3 again show that the dominant species in each CST indeed has the highest specificity value in CST I-III (CST V was not detected in this cohort probably due to limited sample size). In contrast, the specificity values of top 10 species with highest specificity in CST IV-A and IV-B are distributed rather evenly (less aggregated), with a rather narrow range. In addition, the top 10 species with highest specificity values in Type IV-B do contain some BVAB species such as *Atopobium*, which has the highest specificity value (0.908). Both the two subtypes of CST IV have the lowest SAI of 0.015 and 0.043, respectively, much smaller than those of CST I-III. The differences between CST IV and other three CSTs range from 5 to 24 times.

The two subtypes of CST IV show similar pattern of specificity distribution, with IV-B has moderately higher SAI, approximately 3 times that of IV-A. The type IV-B does contain more BVAB (bacterial vaginosis associated bacteria) such as *Atopobium* and *Gardnerella* in the top 10 species with highest specificity, which is consistent with Gajer et al. (2012) characterization.

## Summary

In summary, the integrated utilization of *species specificity* index and *specificity aggregation index* offers an effective quantitative tool to characterize the CSTs in the human vaginal microbial communities. CST I, II, III, and V can be identified with the species of the highest specificity value. CST IV lacks a species with predominantly large specificity value, and instead the specificity values in the CST IV are rather *dispersed* (even or less aggregated). Quantitatively, the SAI of CST IV is approximately 1/5-1/60of the SAI of other types. In addition, as demonstrated below in the ‘Discussion’, CST IV has significantly higher community diversity than the other four CSTs, which can also be harnessed to distinguish CST IV from the other CSTs. But as explained previously, community diversity alone can only distinguish the CST IV from the LDT (the other four types).

Beyond offering a quantitative tool to characterize CSTs, *specificity* also explains the underlying mechanism that shapes the CSTs. From the perspective of specificity spectrum, the five CSTs can be categorized as two classes: the *diverse* class (CST IV) *vs*. LDT class (CST I, II, III, & V). The *diverse* class is characterized by multiple *specialist* species, while the *dominated* class is often characterized by a single *specialist* species. At the ecological time scale, the transitions among the CSTs may be considered as ‘discrete’ manifestations of the human vaginal microbial community dynamics, which are driven by the *host environment* (determined by the host genetic background and diverse biotic and abiotic host factors). The BV-associated dysbiosis may be characterized by the breakup of the normal transitions of CSTs, and therefore quantifiable with the changes of specificity. Therefore, specificity-based analysis of the CST transitions may play an important role in assessing the risk of BV occurrence/recurrence. At the evolutionary time scale, it is the species adaptation that ultimately shapes its distribution and abundance, which determines the species specificity according to Eq. (1). Therefore, CSTs should be the evolved characteristics of the human vaginal microbiome.

In perspective, the advance we made in this article not only facilitate the accurate applications of the CST scheme but also demonstrate a general approach to quantitatively characterizing other microbiomes such as the gut enterotypes. It also offers hints towards an effective classification tool for microbiome-typing, which is not implemented yet in this study.

## Discussion

### Can the diversity profile differentiate the CSTs?

We computed the alpha diversities in the Hill numbers of all 394 communities representing five CSTs, and the computed Hill numbers are listed in Table S3. We further performed the significance test for the difference between any two CSTs, and the results are displayed in Table 4. The significance test demonstrates that the Hill numbers of the CST IV are indeed significantly different from (larger than) those of the other four CSTs across the board of all diversity orders (*q* = 0–4) we tested. However, diversity profile analysis failed to resolve the difference among the other four types. Therefore, relying on diversity analysis alone is not able to distinguish all of the five CSTs.

**Table 4:** The *p*-values of the significance tests for the difference in the alpha diversity (Hill numbers) among five CSTs with the 400-cross-sectional cohort dataset.

Types	*q* = 0	*q* = 1	*q* = 2	*q* = 3	*q* = 4
I *vs*. II	0.001	0.155	0.264	0.318	0.351
I *vs*. III	0.951	0.599	0.426	0.409	0.404
I *vs*. IV	0.000	0.000	0.000	0.000	0.000
I *vs*. V	0.733	0.260	0.236	0.214	0.228
II *vs*. III	0.001	0.093	0.144	0.156	0.167
II *vs*. IV	0.000	0.000	0.000	0.000	0.000
II *vs*. V	0.107	0.793	0.570	0.585	0.526
III *vs*. IV	0.000	0.000	0.000	0.000	0.000
III *vs*. V	0.726	0.106	0.050	0.047	0.046
IV *vs*. V	0.000	0.000	0.000	0.000	0.000

We also computed the alpha diversities (in the Hill numbers) of all 937 community samples from the 32-longitudinal cohort dataset, representing four CSTs (CST V was not detected in the cohort due to the limited numbers of subjects investigated), and the computed Hill numbers were listed in Table S4. We further performed the significance test for the difference between any two CSTs, and the results are displayed in Table 5. The significance test demonstrates that the Hill numbers of the CST IV (including IV-A and IV-B) are indeed significantly different from (larger than) those of the other three CTSs (CST I, II, III) across the board of all diversity orders (*q* = 0–4) we tested. However, the diversity profile analysis again failed to resolve the differences among the other three types. Therefore, relying on diversity analysis alone is not able to distinguish all of the CSTs.

**Figure 1:**
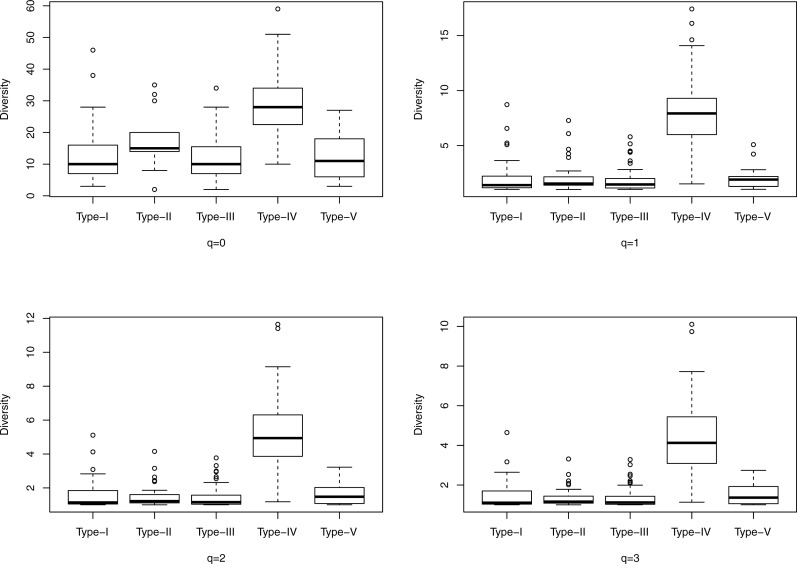
The box plots of the Hill numbers (*y*-axis) corresponding to different CSTs (*x*-axis) based on the information from Table S3 (400-cross-sectional cohort). Each sub-graph corresponds to a different diversity order *q*, ranging from *0* to *3*.

**Table 5:** The *p*-values of the significance tests for the difference in the alpha diversity (Hill numbers) among five CSTs with 32-longitudinal dataset.

Types	*q* =0	*q* =1	*q* =2	*q* =3	*q* =4
I *vs*. II	0.326	0.000	0.000	0.000	0.000
I *vs*. III	0.231	0.000	0.000	0.000	0.000
I *vs*. IV-A	0.000	0.000	0.000	0.000	0.000
I *vs*. IV-B	0.000	0.000	0.000	0.000	0.000
II *vs*. III	0.898	0.000	0.000	0.000	0.000
II *vs*. IV-A	0.000	0.000	0.000	0.000	0.000
II *vs*. IV-B	0.000	0.000	0.000	0.000	0.000
III *vs*. IV-A	0.000	0.000	0.000	0.000	0.000
III *vs*. IV-B	0.000	0.000	0.000	0.000	0.000
IV *vs*. IV-B	0.000	0.000	0.000	0.000	0.000

**Figure 2:**
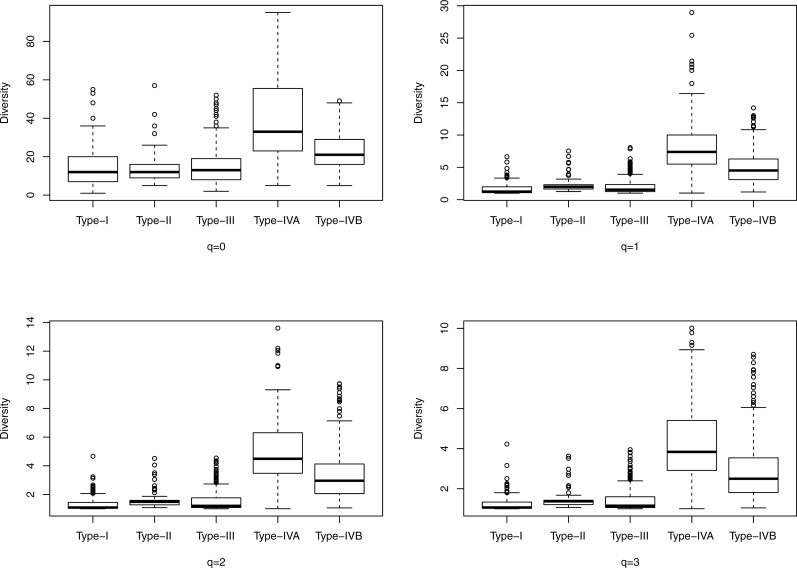
The box plots of the Hill numbers (*y*-axis) corresponding to different CSTs (*x*-axis) based on the information from Table S4 (32-longnitudinal cohort). Each sub-graph corresponds to a different diversity order *q*, ranging from *0* to *3.*

Obviously, as mentioned previously, the significantly high community diversity can also be employed to distinguish between CST IV and other CSTs. Tables 4 and 5 also exhibit an interesting phenomenon that some of the higher order Hill numbers (alpha diversities) beyond order zero (species richness) may display significant differences among CSTs, but there is not a consistent pattern other than the consistently significant difference between CST IV and other CSTs.

Figure 1 shows the box plots of the Hill numbers (*y*-axis) corresponding to different CSTs (*x*-axis) based on the information from Table S3, which contains the alpha diversity in the Hill numbers of the 400-cross-sectional cohort. Each sub-graph corresponds to a different diversity order *q*, ranging from *0* to *3*. The characteristic of significantly high diversity of CST IV, is obvious in Fig. 1. Similarly, Fig. 2 shows the box plots of the Hill numbers based on the information of the 32-longitudinal dataset (Table S4). The characteristics of CST IV-A and IV-B are highlighted in Fig. 2.

### The statistical distribution of specificity

We further characterize the CSTs by fitting two canonical statistical distributions, i.e., the normal distribution and power law distribution, to the diversity and specificity values, respectively. The results for the 400-cross-sectional cohort dataset are listed in Table 6, and those for the 32- longitudinal cohort dataset are listed in Table 7. In both tables, we listed the *p*-values of fitting the normal distribution and power law distribution with the alpha diversity and specificity data. In the case of the power law distribution, we also listed its parameter (*K*), which measures the heterogeneity level of the random variable (diversity or specificity). The higher the *K* value is, the higher the heterogeneity.

**Table 6:** The results of distribution-fittings for the alpha *diversity* and species *specificity* with the 400-cross-sectional cohort dataset.

Types	Parameters	Alpha diversity (Hill numbers)					Specificity
		*q* = 0	*q* = 1	*q* = 2	*q* = 3	*q* = 4	
Type I	Normal (*p-value*)	0.000	0.000	0.000	0.000	0.000	0.000
	Power law (*p-value*)	1.000	0.999	0.997	0.079	0.983	0.992
	Power law (*K*)	5.532	3.943	5.771	3.879	7.375	1.915
Type II	Normal (*p-value*)	0.013	0.000	0.000	0.000	0.000	0.000
	Power law (*p-value*)	0.930	0.942	0.944	0.905	0.879	0.999
	Power law (*K*)	3.977	2.670	3.603	4.209	4.560	2.354
Type III	Normal (*p-value*)	0.000	0.000	0.000	0.000	0.000	0.000
	Power law (*p-value*)	1.000	0.360	0.999	0.723	0.788	0.880
	Power law (*K*)	11.208	3.213	5.531	4.774	5.016	1.799
Type IV	Normal (*p-value*)	0.254	0.104	0.015	0.007	0.004	0.000
	Power law (*p-value*)	0.996	1.000	0.978	0.016	0.022	0.076
	Power law (*K*)	7.439	6.606	7.445	3.623	3.707	1.607
Type V	Normal (*p-value*)	0.121	0.001	0.013	0.016	0.014	0.000
	Power law (*p-value*)	0.560	0.999	0.587	1.000	0.661	0.378
	Power law (*K*)	2.848	4.291	3.302	8.853	3.821	2.465

**Table 7:** The results of distribution-fittings for the alpha *diversity* and species *specificity* with the 32-longitudinal cohort dataset.

Types	Parameters	Alpha diversity (Hill numbers)					Specificity
		*q* = 0	*q* = 1	*q* = 2	*q* = 3	*q* = 4	
Type I	Normal (*p-value*)	0.000	0.000	0.000	0.000	0.000	0.000
	Power law (*p-value*)	1.000	1.000	0.767	0.824	0.817	0.291
	Power law (*K*)	4.956	5.576	3.949	4.413	4.767	1.574
Type II	Normal (*p-value*)	0.000	0.000	0.000	0.000	0.000	0.000
	Power law (*p-value*)	1.000	0.895	0.749	0.089	0.071	0.086
	Power law (*K*)	3.327	3.185	3.521	4.908	5.287	1.316
Type III	Normal (*p-value*)	0.000	0.000	0.000	0.000	0.000	0.000
	Power law (*p-value*)	0.976	0.472	0.051	0.045	0.966	0.407
	Power law (*K*)	4.464	2.844	3.461	3.666	6.571	1.493
Type IV-A	Normal (*p-value*)	0.000	0.000	0.000	0.000	0.000	0.000
	Power law (*p-value*)	0.464	0.771	0.935	0.759	0.794	0.779
	Power law (*K*)	2.723	3.014	3.864	3.869	4.154	2.635
Type IV-B	Normal (*p-value*)	0.000	0.000	0.000	0.000	0.000	0.000
	Power law (*p-value*)	0.981	0.483	0.616	0.351	0.565	0.052
	Power law (*K*)	10.918	3.439	3.322	3.417	3.511	1.384

**Figure 3:**
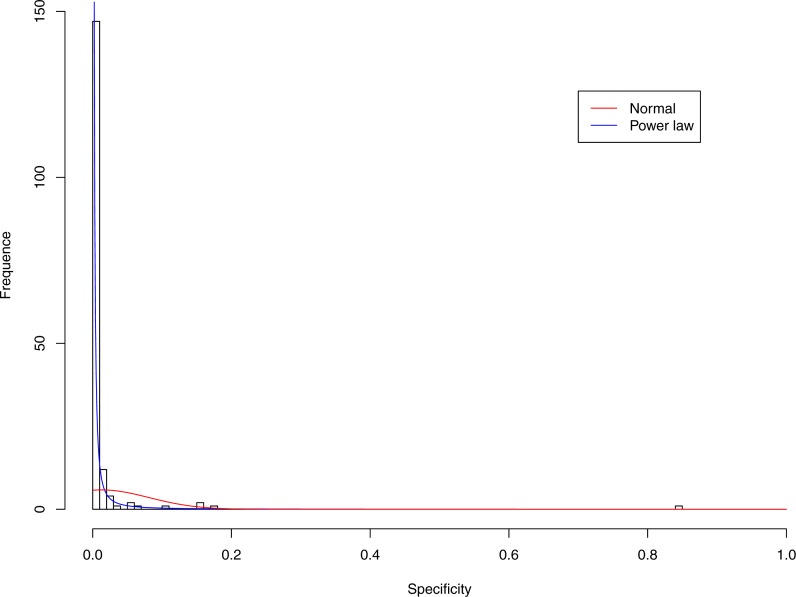
Fitting power law distribution and normal distribution to the specificity of CST-I: the power law distribution (green curve) succeeded, while the normal distribution failed.

**Figure 4:**
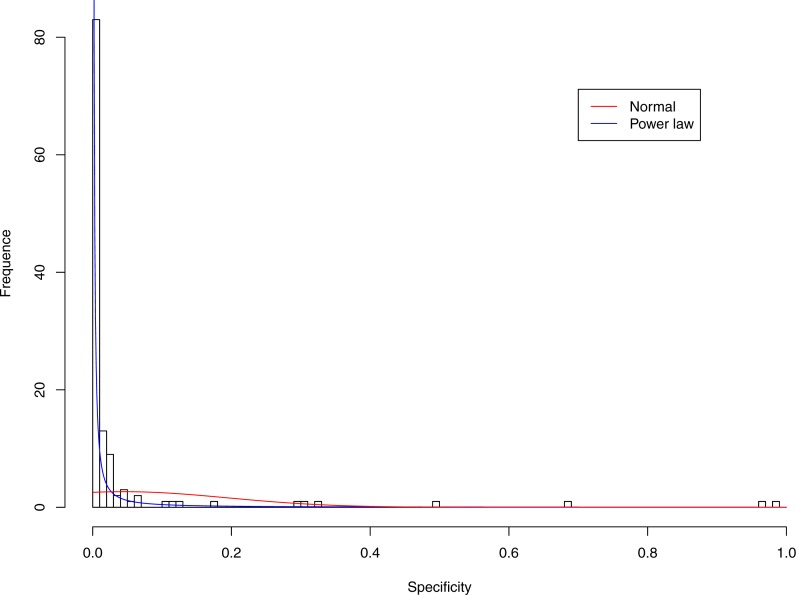
Fitting power law distribution and normal distribution to the specificity of CST-II: the power law distribution (green curve) succeeded, while the normal distribution failed.

**Figure 5:**
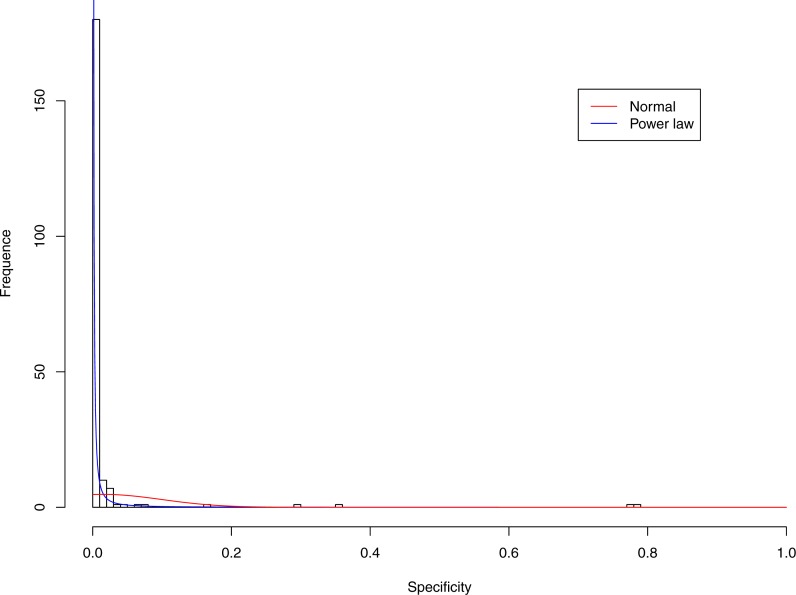
Fitting power law distribution and normal distribution to the specificity of CST-III: the power law distribution (green curve) succeeded, while the normal distribution failed.

**Figure 6:**
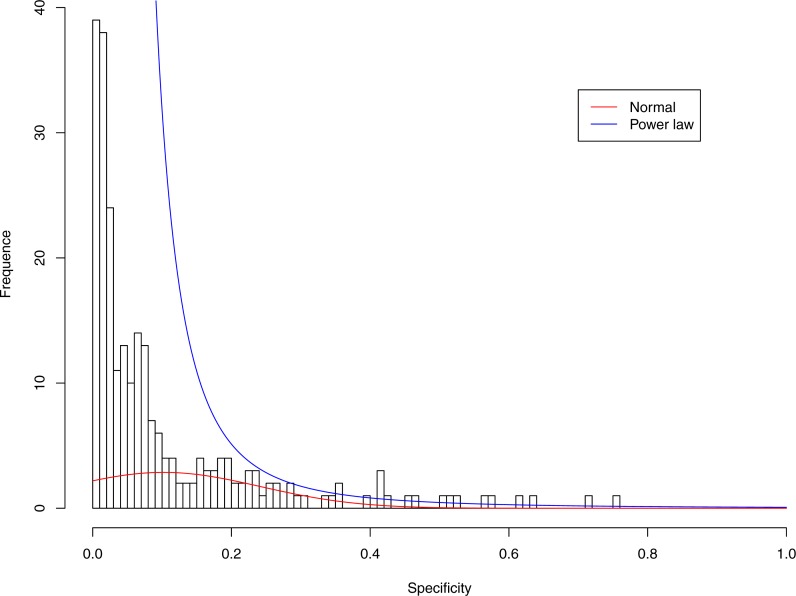
Fitting power law distribution and normal distribution to the specificity of CST-IV-A: the power law distribution (green curve) succeeded, while the normal distribution failed.

**Figure 7:**
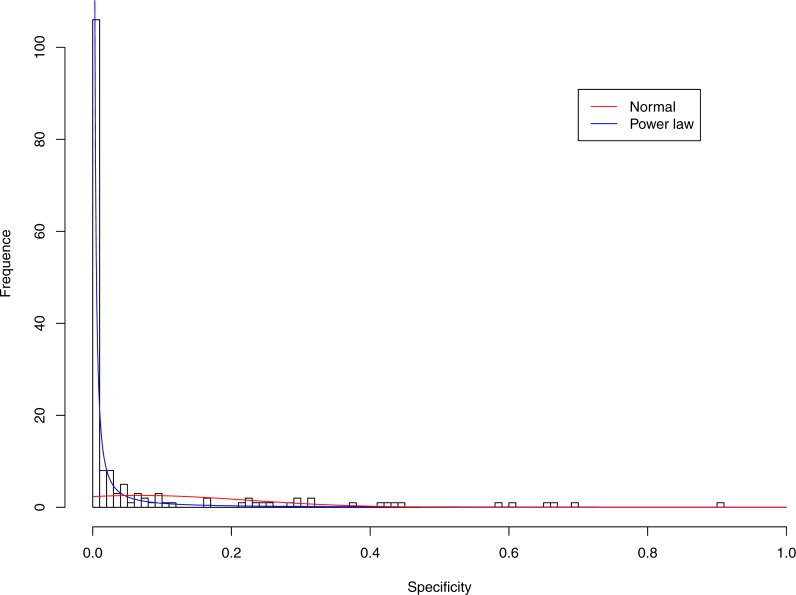
Fitting power law distribution and normal distribution to the specificity of CST-IV-B: the power law distribution (green curve) succeeded, while the normal distribution failed.

Tables 6 and 7 show that, in most cases, the normal distribution failed to describe the distributions of diversity or specificity. Among 60 tested cases, in only two cases the normal distribution succeeded in fitting the diversity at order zero of CST IV and V (*p*-value > 0.1). We consider the two cases as exceptions and conclude that neither diversity nor specificity fits to the normal distribution. In contrast, the power law distribution successfully fitted to all 60 but 4 cases. The prevalence of the power law distribution suggests that the distribution of the diversity or specificity is highly skewed: (i) a few community samples display disproportionally high diversity, while most community samples display low diversity in the case of diversity distribution; (ii) a few species display disproportionally high specificity, while most species display low specificity.

Figures 3–7 display the fitting of the power law and normal distributions for each CST with the 32-longnitudinal-cohort dataset. In the graphs for all five CSTs, power law distribution fits to the specificity distribution data well, while normal distribution fail to fit to the specificity of any CST.

It is particularly worthy of noting that the only 4 failures of the power law distribution in fitting specificity occurred in CST IV. That is, the distribution of specificity in CST IV is far less aggregated or far more even than in other CSTs, and therefore does not fit to the power law distribution. The finding here is obviously consistent with the previous discussed characteristics of CST IV, which lacks predominant species, and maintains a far smaller specificity aggregation index than the other CSTs.

##  Supplemental Information

10.7717/peerj.3366/supp-1 Supplemental Information 1 Supplementary Tables S1–S4 **Table S1**. Species specificity list of the five CSTs from 400 cross-sectional cohort.
**Table S2**. Species specificity list of the five CSTs from 32-longitudinal cohort.
**Table S3**. The alpha diversity (Hill numbers) of the five CSTs from 400-cross-sectional cohort.
**Table S4**. The alpha diversity (Hill numbers) of the five CSTs from 32-longitudinal datasets.

## Additional Information and Declarations

### Data Availability

The following information was supplied regarding data availability: The datasets of 400-cross-sectional cohort and 32-longitudinal cohort are available in the online supporting information of Ravel et al. (2011) (http://www.pnas.org/content/suppl/2010/06/03/1002611107.DCSupplemental) and Gajer et al. (2012) (http://www.ncbi.nlm.nih.gov/pmc/articles/PMC3722878/#S19title).
